# Structural, Photophysical, and Electronic Properties of CH_3_NH_3_PbCl_3_ Single Crystals

**DOI:** 10.1038/s41598-019-49926-z

**Published:** 2019-09-16

**Authors:** Hao-Ping Hsu, Liang-Chen Li, Muthaiah Shellaiah, Kien Wen Sun

**Affiliations:** 1 Department of Applied Chemistry, National Chiao Tung University, 1001 University Road, Hsinchu, 30010 Taiwan; 2 Center for Nano Science and Technology, National Chiao Tung University, Hsinchu, 30010 Taiwan

**Keywords:** Chemistry, Materials science

## Abstract

Methylammonium lead chloride (CH_3_NH_3_PbCl_3_ or MAPbCl_3_) single crystals were fabricated using the inverse temperature crystallization method, and their structural, photophysical, and electronic characteristics were studied using temperature dependent optical spectroscopy, X-ray diffraction (XRD), current-voltage, and Hall measurements. The changes in absorption and photoluminescence properties accompanied with structural changes in crystal lattice were studied within a broad temperature range of 300–20 K. XRD investigations reveal that phase changes took placed around 180 K and 175 K. At a temperature below 170 K, two different crystallographic phases were found to co-exist in the photoluminescence spectra. An asymmetric line shape with broad and weak shoulders near the absorption edges was observed in all of the major PL peaks. The weak shoulders are attributed to the missing chloride atoms on the crystal surface. The photoluminescence intensity of the crystals was strongly influenced by the environment, thereby indicating that the carrier recombination is affected by the physical desorption/absorption of gas molecules at the crystal surface. Moreover, vibronic replicas in the photoluminescence spectra at low temperature were observed for the first time. The origins of these replicas are attributed to the coupling between the vibrational/librational motions of the organic cations and the photoexcited electrons. Finally, the Hall and current-voltage measurements confirm that the crystal is an n-type semiconductor with a carrier concentration of ~2.63 × 10^11^ cm^−3^, a mobility of 4.14 cm^2^/V•s, and a conductivity of 1.8 × 10^−8^ *Ω*^−1^ *cm*^−1^ under dark and room temperature conditions.

## Introduction

Organic/inorganic hybrid methylammonium lead halide perovskite (MAPbX_3_) is the most promising energy materials for photovoltaic and optoelectronic applications. Altering the properties of these materials for improvement is obtained by varying the type of metallic or halide ions^1^. MAPbX_3_ solar cells have progressed in efficiency faster than any other solar cells since their invention^2–5^. Researches in metal-halide perovskites as promising optoelectronic materials for solid-state light emitting applications^6–8^ and detectors^9,10^ beyond photovoltaics have increased. Metal-halide perovskites are inexpensive solution-processable materials with excellent intrinsic properties that render them appropriate candidates for technologies in the future. However, commercialization of perovskite devices is hindered by rapid material degradation^11,12^, hysteresis^13,14^, and environmental factors, such as moisture and heat^15^. Even with the outstanding materials properties and device performances of hybrid perovskites, some restrictions must be solved in order to gain intact optoelectronic and sensing applications and commercialization. Understanding the underlying mechanisms behind is necessary to analyze the material and enhance the efficiency, sensitivity, and stability of devices based on these perovskites.

Previous researches focused mostly on polycrystalline perovskite thin films^16–22^ for characterizing their material properties. However, the intrinsic electronic or optoelectronic properties of polycrystalline thin films are overshadowed by the micro-, nano- and/or non-crystalline domains. On the other end of the spectrum, perovskite single crystals are an ideal platform for investigating the intrinsic structural and photophysical properties of perovskites because they are free from grains and amorphous domains, thereby improving the efficiency and long-term stability of polycrystalline perovskite optoelectronic and photovoltaic devices.

Compared to perovskite thin films, reports on MAPbX_3_ single crystals are relatively few. For instance, MAPbI_3_ and MAPbBr_3_ single crystals show carrier diffusion lengths over 100 μm and high hole and electron mobilities of 9400 and 2800 cm^2^-V^−1^s^−1^, respectively^23–25^. α–phase CH_3_NH_3_PbBr_3_ bulk crystals studied by spectroscopic ellipsometry^26^ from 0.73–6.45 eV showed a strong optical transition at ~2.3 eV and randomly oriented cations at room temperature. Low trap density and exceptionally long and balanced carrier diffusion lengths were reported in decent-sized MAPbX_3_ single crystals^27^. The dynamics of photoexcited carriers, such as recombination mechanisms in surface or bulk and carrier diffusion in MAPbBr_3_ single crystals, was studied using transient optical spectroscopy^28–30^. MAPbBr_3_ single crystals in the orthorhombic phase were reported to have an exciton binding energy of 15.33 meV and a Bohr radius of ~4.38 nm at a low temperature by Tilchin *et al*.^31^. The optical band gap, carrier recombination, excitation spectra, PL spectral position, and lifetime characteristics of the incipient surface of a MAPbBr_3_ single crystal are different from that of a pristinely cleaved crystal surface^32^. More recently, structural and photophysical properties of MAPbBr_3_ single crystals were studied using temperature dependent optical and X-ray diffraction (XRD) techniques^33^. A direct time domain view of large polaron formation in MAPbBr_3_ single crystals was provided by Miyata *et al*.^34^, who revealed that, irrespective of the cation type, the large polaron is formed mainly from the deformation of the PbBr_3_^−^ frameworks.

Despite lead halide perovskite single crystals have been intensively researched, methylammonium lead chloride (MAPbCl_3_) as an important member in perovskite family, attracts less attention. As a wide bandgap semiconductor, the MAPbCl_3_ is transparent to visible but responsive to UV radiation; its absorption, which is mainly limited to wavelengths no longer than 400 nm, makes it a suitable candidate for UV applications. These wide bandgap perovskite semiconductors are promising candidates for solution-processed UV light emitting devices, photodetectors, and transparent electronics. For example, UV photodetectors based on crystalline MAPbCl_3_ exhibited improved figures of merit by several orders of magnitude^35,36^. Photo response from metal-semiconductor-metal detectors based on MAPbCl_3_ single crystals showed dependence on the crystal orientations^37^. Future device refinement and advancement of MAPbCl_3_-based devices could be possible only by further exploring their intrinsic properties, such as a better understanding in crystal structures/energy levels and mechanisms in charge transport and separation. Despite laborious research, opinions regarding these basic properties are inconsistent, and more investigations are required to resolve these dissents.

This study aims to distinguish the intrinsic structural, photophysical, and electronic characteristics between MAPbCl_3_ single crystals and polycrystalline thin films by focusing on MAPbCl_3_ single crystals synthesized from solutions, which show less defects and reduced grains. These material properties were investigated using temperature dependent single crystal/powder XRD, continuous wave photoluminescence (PL), absorption/transmittance, Raman spectroscopy, and Hall and current-voltage (I–V) measurements at a wide temperature range.

## Experimental Methods

### General Information

The crystal structures and phase transitions of the as-grown MAPbCl_3_ single crystals were investigated by temperature dependent XRD. Single crystal and powder XRD studies were conducted with a Bruker D8 Discover X-ray Diffraction System from 300–20 K. Transmission electron microscopy (TEM) samples and high-resolution TEM (HRTEM) images were obtained using a TESCAN LYRA 3 Dual-Beam Focus Ion Beam Microscope and a JEOL-JEM-2100F, respectively. Temperature dependent absorption/transmittance spectra from room temperature to 20 K were taken using a combination of a HOROBA iHR-550 spectrometer, Xenon lamp, and liquid-nitrogen cooled CCD detector with the samples placed in a thermostat. Temperature dependent continuous wave PL spectra were recorded at 300–20 K with a HOROBA iHR-550 spectrometer, liquid-nitrogen cooled CCD detector, and semiconductor laser operated at 266 nm. A Lab RAM HR instrumental setup using a HOROBA iHR-550 spectrometer and a 532 nm semiconductor laser were employed to conduct Raman investigations at room temperature.

Hall measurements were taken in vacuum with a GMW model 5430 instrument at room temperature with a sample size of ~2.5 mm × 2 mm × 1 mm at a scanned magnetic field up to 0.375 Tesla. The current-voltage (I–V) curves were recorded with an EverBeing model CG-196 two-point and four-point probe station at room temperature with sample placed in dark and/or illuminated with a 405 nm laser.

### Synthesis of MAPbBr3 single crystal

The inverse temperature crystallization (ITC) method reported in an earlier study^24^ was adapted to prepare the MAPbBr_3_ single crystals from a solution. Lead chloride (PbCl_2_, 99.999%, Alfa Aesar), methylammonium hydrochloride (CH_3_NH_3_Cl, 99%, Alfa Aesar), dimethylformamide (DMF) (C_3_H_7_NO, 99.5%, Merck KGaA), and dimethyl sulfoxide (DMSO) (C_2_H_6_OS, 99.7%, Sigma-Aldrich) were used as received and without further purification. A total of 0.2228 g of CH_3_NH_3_Cl were added quickly to 3.3 mL DMSO:DMF (1:1) solution in an ultrasonic bath under a N_2_ atmosphere at room temperature for 10 min until the CH_3_NH_3_Cl was totally dissolved. Then, 0.8343 g PbCl_2_ was added to 3 mL CH_3_NH_3_Cl/DMSO:DMF (1:1) solution and stirred for 20 min until the solution became transparent. The solution was filtered using PVDF filter. The filtrate was placed in a vial and kept in an oil bath undisturbed at 50 °C for 6 ~8 h till single crystals formed in a size of ~2.5 × 2 mm with a thickness of ~1 mm. Once millimeter-sized single crystals were formed, they were taken out from the vial and dried with a nitrogen gun.

## Results and Discussions

**Figure 1:**
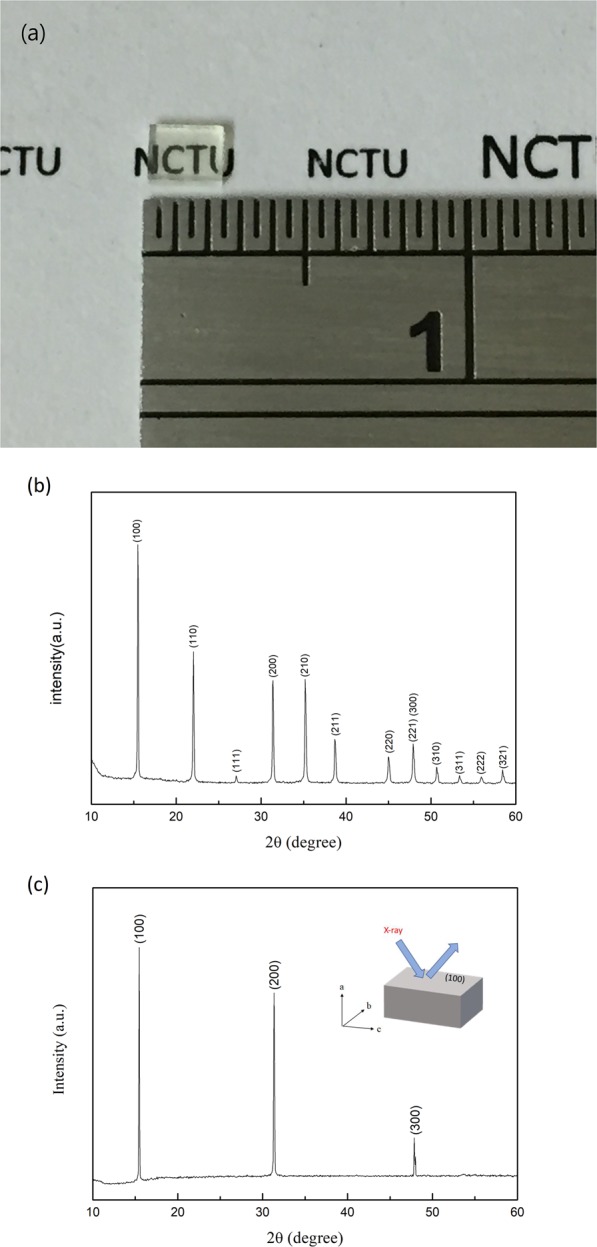
(**a**) Optical image of the MAPbCl_3_ single crystal prepared by the modified ITC method (**b**) powder and (**c**) single crystal XRD patterns of the crystal at room temperature. The inset illustrates the crystal orientation during XRD measurements.

The optical image of a MAPbCl_3_ single crystal prepared by the modified ITC method with dimensions of ~2.5 × 2 × 1 mm^3^ is shown in Fig. 1(a). Figure 1(b,c) display the obtained powder and single crystal XRD spectra at room temperature, respectively. The crystal adopted the centrosymmetric ${Pm}\bar{3}m$ cubic space group at 300 K. The diffraction peak positions (Fig. 1(b)) at 15.71°, 22.22°, 27.24°, 31.58°, 35.38°, 38.86°, 45.18°, 48.09°, 50.85°, 53.52°, 56.07°, and 58.58° were transformed into interplanar distances, which correlated to the (100), (110), (111), (200), (210), (211), (220), (221)(300), (310), (311), (222), and (321) crystal planes, respectively. These results agree with earlier reports^36^. Due to the immediate amorphization or liquidation of the material when exposed to the high energy electron beam, TEM images or selective area electron diffraction (SAED) patterns of the crystal cannot be obtained. The TEM/SEM images of the sample slices prepared with focused ion beam are shown in Fig. S1 in Supporting Information.

**Figure 2:**
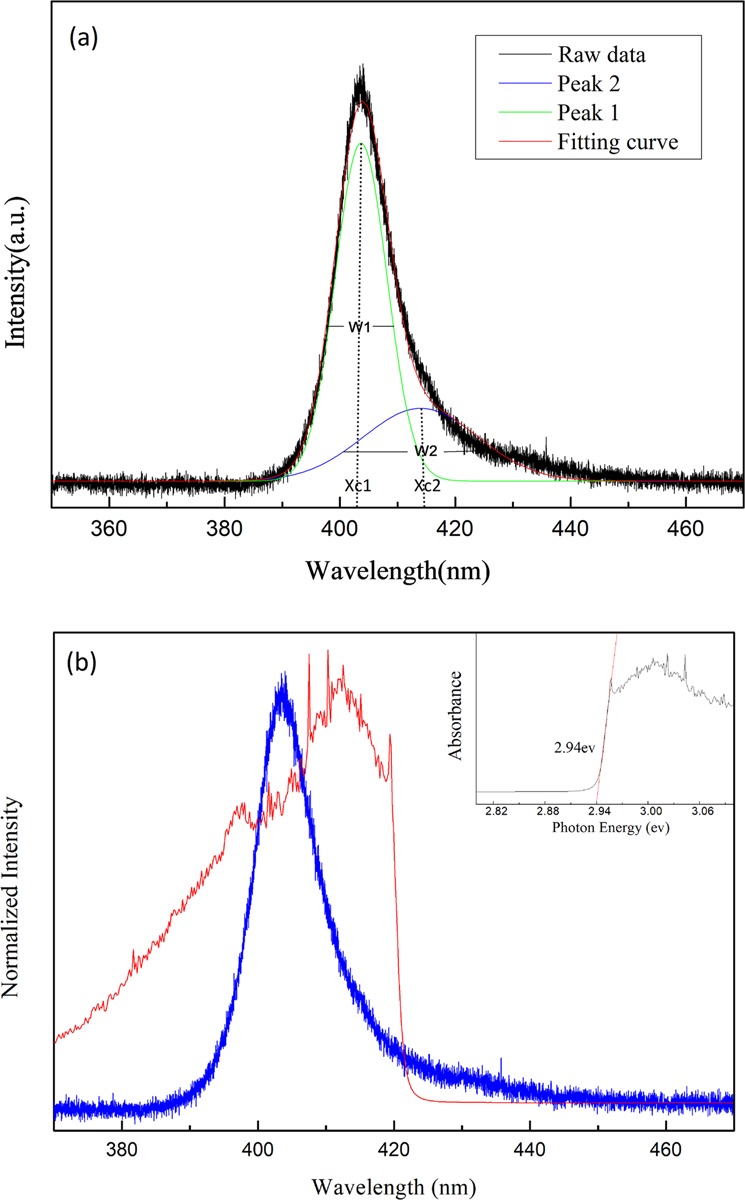
(**a**) PL spectrum fitted with peaks 1 and 2 at 300 K. X_c1_, X_c2_, W_1_, and W_2_ represent the peak positions and bandwidths at FWHM of peaks 1 and 2, respectively. (**b**) PL and UV-Vis absorption spectra of the MAPbCl_3_ single crystal excited at 266 nm. The inset shows the Tauc-plot fitting to determine the onset of the absorption.

The UV-visible PL and absorption spectra of the solution prepared MAPbCl_3_ single crystal excited with a 266 nm semiconductor laser at 300 K are displayed in Fig. 2. Similar to the previous reports for MAPbBr_3_ and MAPbI_3_^25,30,33,38^, the PL spectrum was found to be asymmetric in line shape and trailed toward long wavelengths. The line shape of the spectrum can be fitted by two luminescence peaks (Fig. 2(a)). The major emission peak 1 above the absorption edge arose from the interband transition and was at 404 nm (~3.07 eV) with a linewidth of ~11 nm (FWHM). The weaker emission peak 2 with a broad linewidth of 24 nm at 415 nm (~2.98 eV) was resulted from the recombination of photoexcited carriers in defects (Cl vacancies on the crystal surface) below the energy gap. The inset in Fig. 2(b) shows a bandgap of ~2.94 eV using Tauc-plot. Similar to the previous reports in single crystal perovskite^23,27^, the mission energy of peak 1 was found to be above the bandgap. Be noted that the absorption lineshape could be affected substantially in a thick sample with strong absorption. Moreover, using Tauc-plot for determining bandgap tends to underestimate the real bandgap value due to the tail absorption^39^. Therefore, to yield an accurate bandgap, the sample thickness needs to be controlled within a few microns.

**Figure 3:**
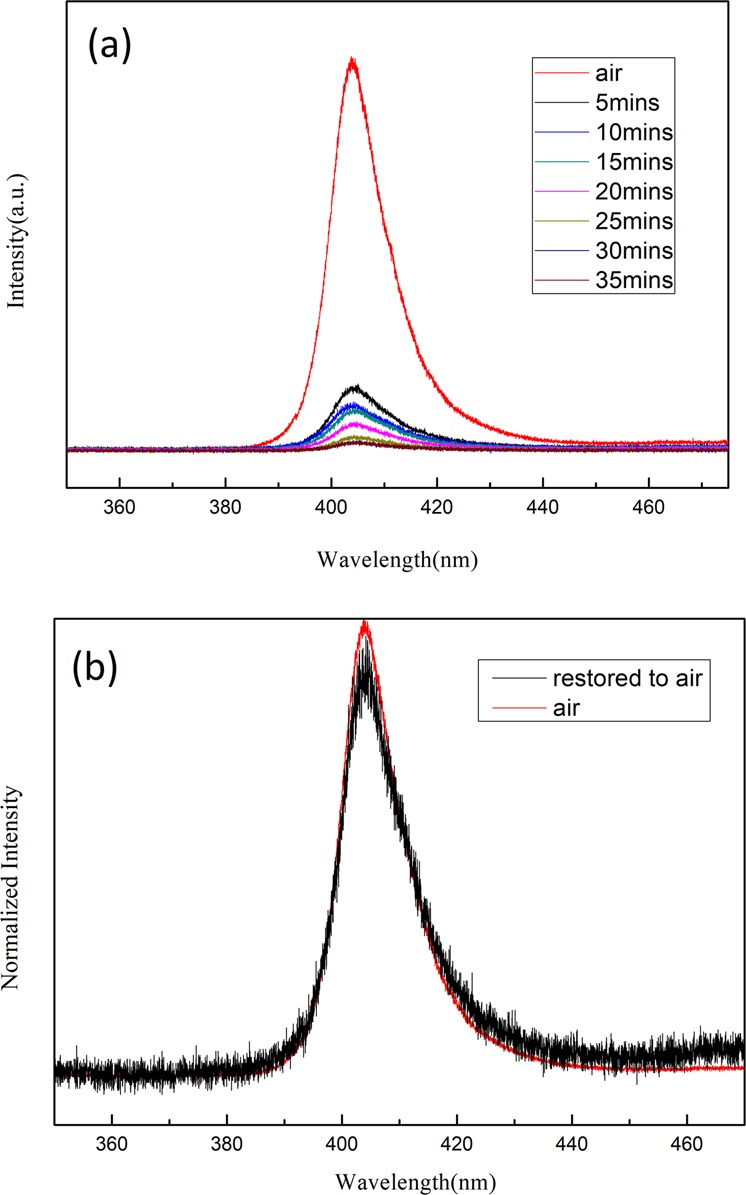
(**a**) PL spectra excited at 266 nm at 300 K under ambient and vacuum. The chamber pressure reached ~10^−3^ torr after pumping down for 35 mins. (**b**) Emission intensity was fully recovered after chamber pressure restored to air.

We then studied the PL intensity behavior of peak 1 to find out the environmental effect on the photogenerated carriers in the MAPbCl_3_ single crystals. The recorded PL spectra excited by a 266 nm semiconductor laser at 300 K in ambient and vacuum (~10^−3^ torr after pumping down for 35 mins) are shown in Fig. 3(a). The PL intensity began to drop when the chamber pressure was lowered toward 10^−3^ torr. However, the PL intensity was fully recovered when the chamber pressure was restored as shown in Fig. 3(b). This result suggested that the carrier recombination is possibly affected by the physical desorption/absorption of gas molecules on the crystal surface. Therefore, the surface of the MAPbCl_3_ single crystal is sensitive to the environment. Recent investigations also indicated that the interplay between hybrid perovskites and environments considerably affect the material’s morphological properties or photostability and its optoelectronic properties^23,29,30,33,40,41^.

**Figure 4:**
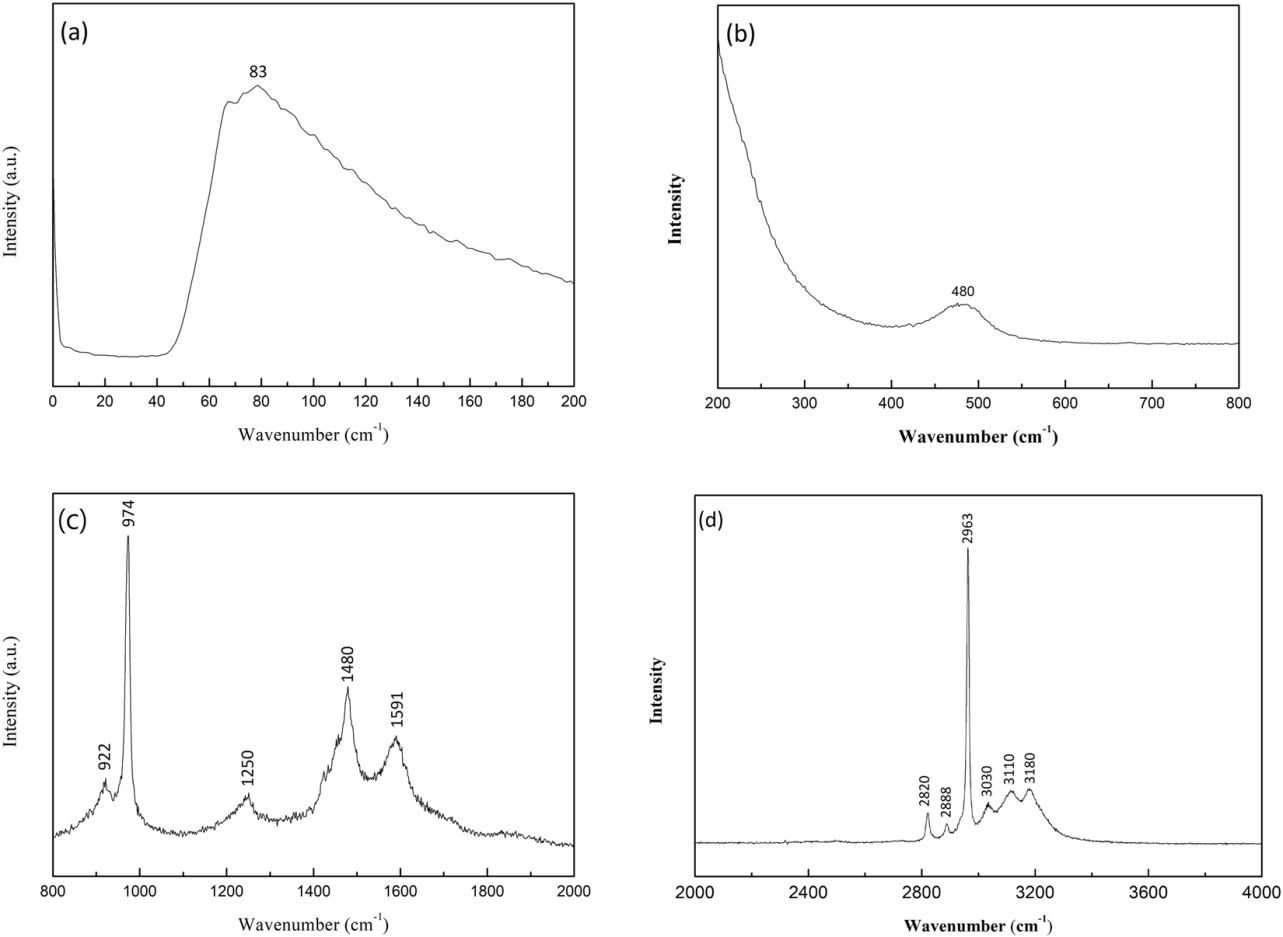
Raman spectra of MAPbCl_3_ single crystals excited at 532 nm at room temperature cover from (**a**) 0–200 cm^−1^ (**b**) 200–400 cm^−1^ (**c**) 800–2000 cm^−1^ and (**c**) 2000–4000 cm^−1^.

To prevent the effect of fluorescence background, a 532 nm semiconductor laser was used in the Raman measurements to study the interaction between the MA^+^ cation and the PbCl_6_^−^ in the octahedral framework. Figure 4(a–d) shows the Raman spectra recorded with a high signal-to-noise ratio and a wavenumber range of 0–4000 cm^−1^ from a MAPbCl_3_ single crystal. In Fig. 4(a), the Raman band at a frequency of 83 cm^−1^ can be attributed to the artifact from the filter cutoff^42,43^. However, the Raman peak at 480 cm^−1^ shown in Fig. 4(b) was corresponded to the limited rotation of the cation in MAPbCl_3_. The middle- and higher-energy peaks displayed in Fig. 4(c,d) were all associated to the various types of the MA^+^ movements. For instance, the distinct and sharp peaks at 922 cm^−1^ and 974 cm^−1^ were originated from the CH_3_NH_3_^+^ rocking and C-N stretching, correspondingly. The intense peak at 2963 cm^−1^ was associated to the symmetric stretching of CH_3_. The assignments of Raman peaks from MAPbCl_3_ single crystals, which are in good agreement with earlier reports^44–46^, are summarized in Table 1. For the lead halide perovskites with atoms of electronegativity strengthens from weakest (I) to strongest (Cl), although the magnitude of the Raman peaks was nearly unchanged, nontheless, either blueshift or redshift in energy could be detected for Raman modes^33,44–46^. These observations indicated that substituting halide atoms with high electronegativity strengthens significantly modifies the microenvironment of the PBX_3_^−^ framework.

**Table 1:** The measured Raman peaks of the MAPbCl_3_ single crystal and assignment of corresponding vibration modes.

MAPbCl_3_ (cm^−1^)	Peak assignment
480	CH_3_NH_3_^+^ torsion
922	CH_3_NH_3_^+^ rocking
974	C–N stretching
1250	CH_3_-NH_3_^+^ rocking
1480	asym. NH_3_^+^ bending
1591	NH_3_^+^ twisting
2820	N^+^–H stretching
2888	Asym. CH_3_ stretching
2963	Sym. CH_3_ stretching
3030	NH_3_^+^ Sym. stretching
3110	
3180	

The temperature dependent structural properties of the MAPbCl_3_ single crystal were analyzed based on temperature dependent absorption/PL spectra and XRD measurements from 300 K to 20 K. Fig. S2(a,b) in Supporting Information display the transitions of the powder and single crystal XRD patterns of the crystal recorded from 300–20 K, respectively. The crystal underwent two structural changes, which associated to the first phase transition from cubic to tetragonal (at temperatures between 200–180 K) followed by a second phase transistion from tetragonal to orthorhombic (at temperatures between 175–170 K). These phase changes were occurred simutaneously with shifts in energy and changes in line shape in the absorption and PL spectra (Fig. S3 in Supporting Information). In the following sections, we categorized and discussed in detail the temperature dependent structural transitions in three temperature ranges as follows: 300 K to 200 K, 200 K to 170 K, and 160 K to 20 K.

### 300 K to 200 K

**Figure 5:**
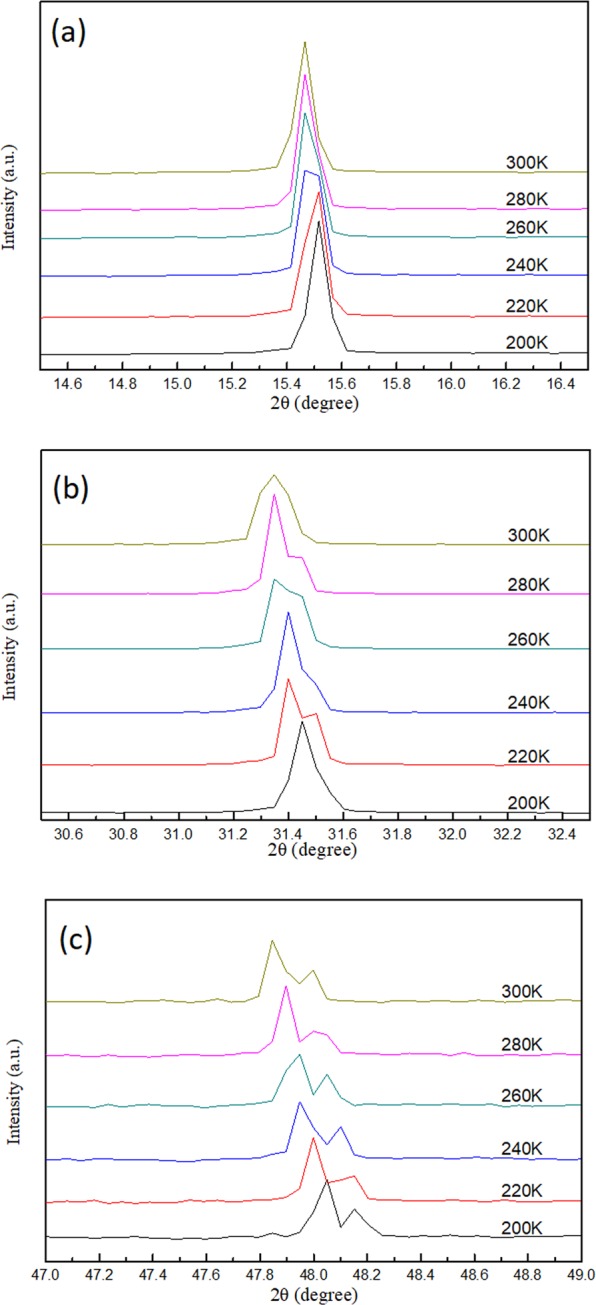
Diffraction spectrum of (**a**) (100) (**b**) (200) and (**c**) (300) peak from single crystal XRD measurements of MAPbCl_3_ single crystal at 300–200 K.

**Figure 6:**
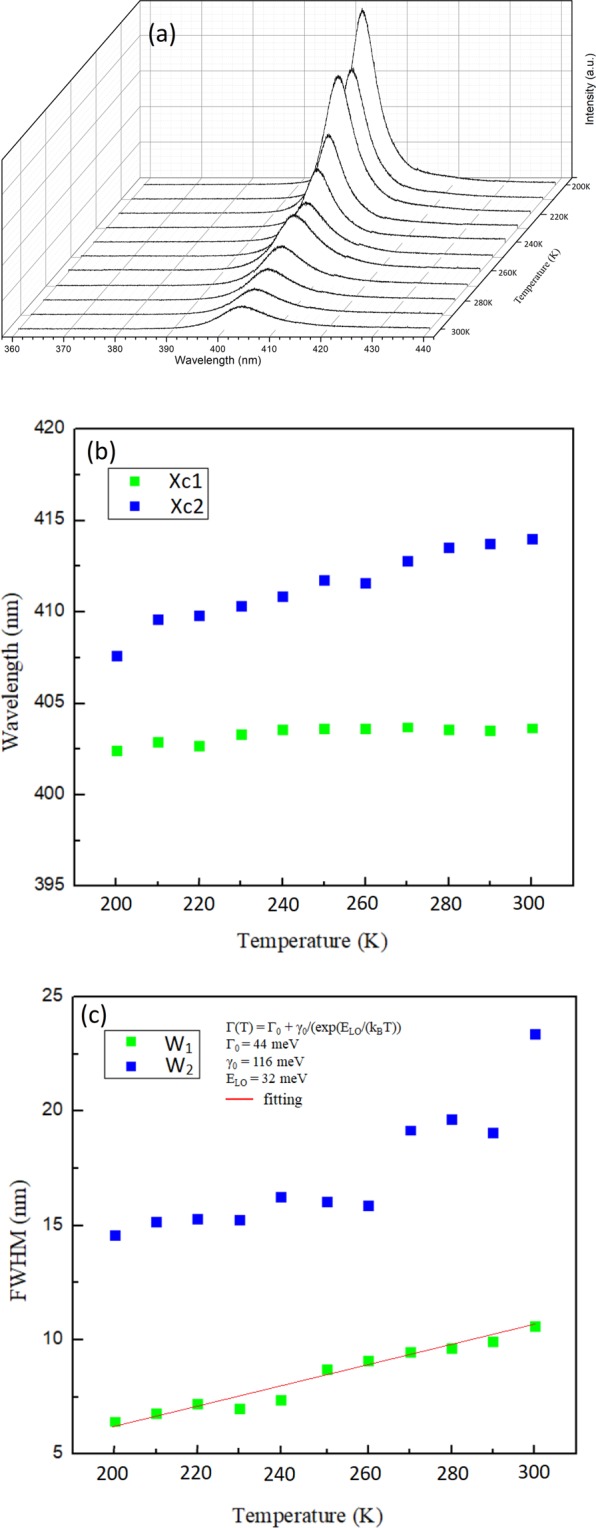
(**a**) Temperature dependent PL spectra of MAPbCl_3_ single crystal excited at 266 nm at 300–200 K. The PL spectra were fitted with peaks 1 and 2 as in Fig. 2(a). X_c1_, X_c2_, W_1_, and W_2_ represent the peak positions and bandwidths at FWHM of peaks 1 and 2. Temperature dependent (**b**) peak positions (X_c1_, X_c2_) and (**c**) bandwidths (W_1_, W_2_) of peaks 1 and 2. The red-colored fitting curve in (**c**) is obtained by using the formula displayed in the inset.

Figure 5(a–c) display the single crystal XRD patterns of (100), (200), and (300) crystal planes when the sample was cooled from 300 K to 200 K. As the temperature was decreased, all diffraction patterns moved toward large diffraction angles because of the lattice contraction. No considerable shift in the diffraction angle was observed for all peaks within this temperature range, thereby indicating that the crystal remained in the cubic phase during cooling. The PL spectra shown in Fig. 6(a) remained asymmetric in line shape when the temperature was lowered to 200 K. The peak positions and full width at half maximum (FWHM) were retrieved by fitting of the PL peaks. Figure 6(b,c) show the changes in the PL peak position and line width as a function of temperature. The FWHM became narrower and the emission intensity of peaks 1 and 2 increased when the temperature decreased. The blueshift in peak 2 was slightly enlarged when the temperature was lowered to 200 K. The temperature dependent FWHM of emission peak 1 that correspond to the cubic phase (Fig. 6(c)) is fitted by considering the temperature inhomogeneous broadening (Γ_o_) and the interaction between LO phonons and photoexcited carriers, described by the Fröhlich Hamiltonian^47^. The inset in Fig. 6(c) shows the extracted fitting parameters with values of Γ_o_ = 44 meV, LO phonon-photoexcited carrier coupling strength γ_o_ = 116 meV, and LO phonon energy E_LO_ = 32 meV.

### 200 K to 170 K

**Figure 7:**
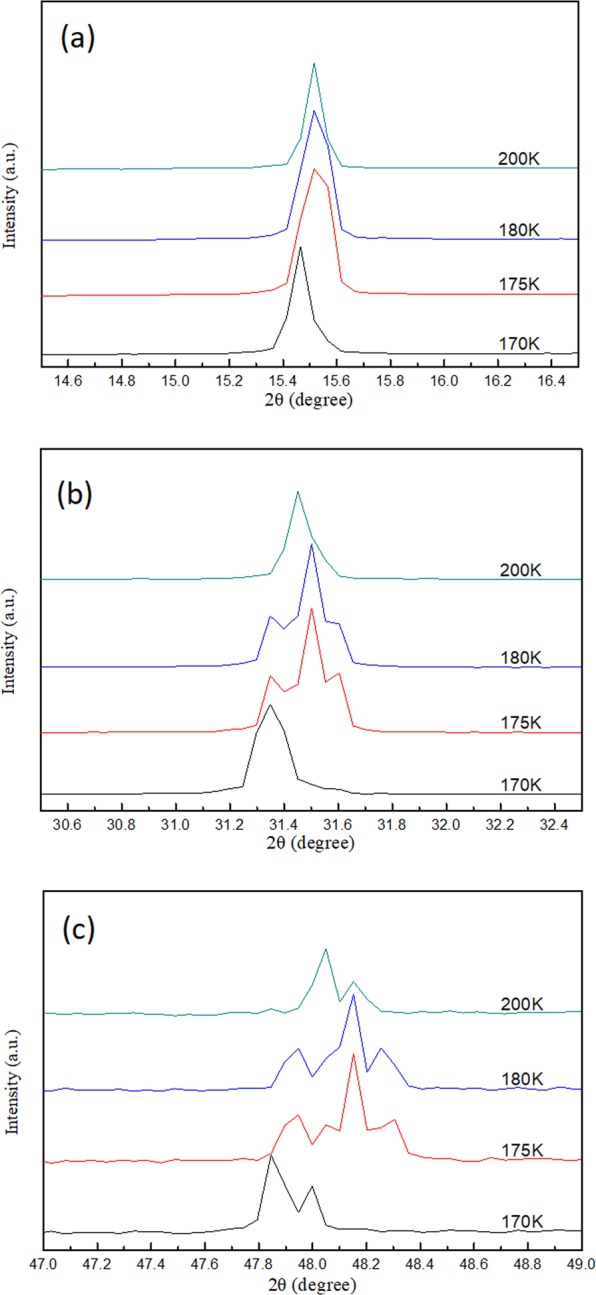
Diffraction spectrum of (**a**) (100) (**b**) (200) and (**c**) (300) peak from single crystal XRD measurements of MAPbCl_3_ at 200–170 K.

When the sample was cooled from 200 K to 170 K, two phase transitions occurred in the MAPbCl_3_ crystal, from cubic to tetragonal (first phase transition) and tetragonal to orthorhombic (second phase transition), as evidenced by the larger shift in diffraction angle and line shape change at 200 K to 180 K and 175 K to 170 K in the XRD spectra shown in Fig. 7(a–c). The first phase transition was majorly induced by the rotational motion around the c-axis of the PbCl_6_^4+^ octahedron^48,49^. The second phase transition was activated when the PbCl_6_^4+^ octahedron inclined out of the ab plane^50^. An illustration of the 3D MAPbCl_3_ structure at different crystal phases displayed in Fig. S4 gives a scenario of the structure changes. Figure S5(a) shows the calculated plane spacing (d-spacing) of the (100) planes using the Bragg diffraction law as a function of temperature from 300 K to 180 K (in cubic phase) shown in the XRD data in Fig. 5(a). Therefore, the thermal expansion coefficient of ~2.44 × 10^−4^ K^−1^ of the crystal can be obtained by fitting the slope of the curve in Fig. S5(a). If no phase transition occurs, then the predicted position of the (100) diffraction peak at 170 K should take placed at an angle of 15.55 degree according to the retrieved thermal expansion coefficient. However, the measured (100) peak was at 15.46 degree (see Fig. S5(b)), which is different from the predicted value. Therefore, we infer that phase transitions occurred at this temperature range.

**Figure 8:**
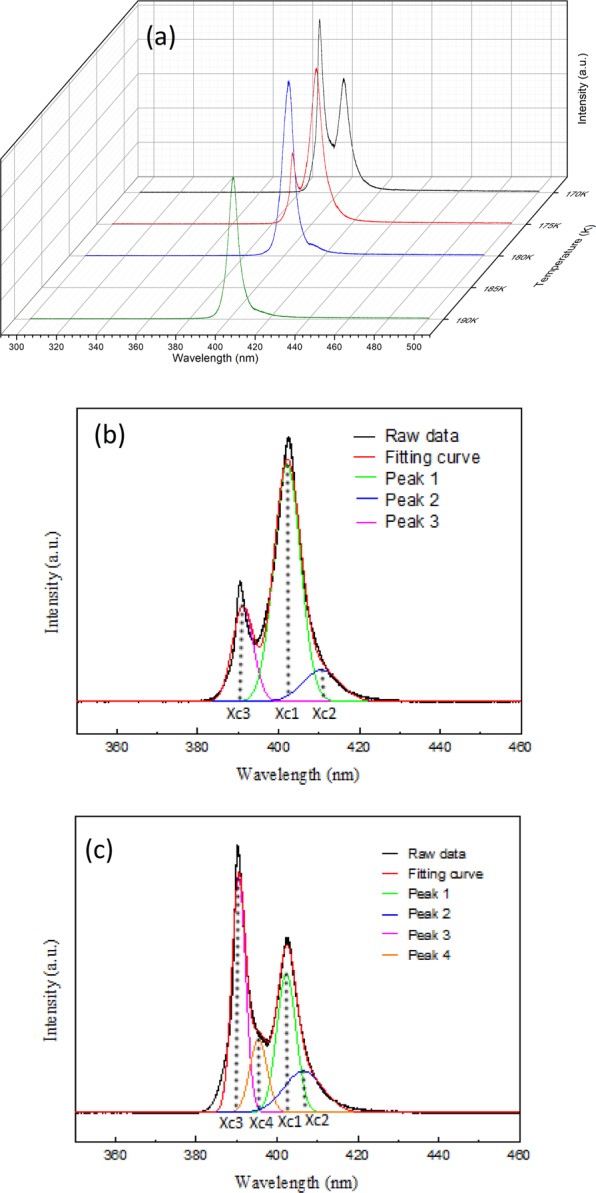
(**a**) PL spectra of MAPbCl_3_ single crystals excited at 266 nm at 190–170 K. (**b**) Fitting of the representative PL spectrum recorded at (**b**) 175 K and (**c**) 170 K in this stage.

**Figure 9:**
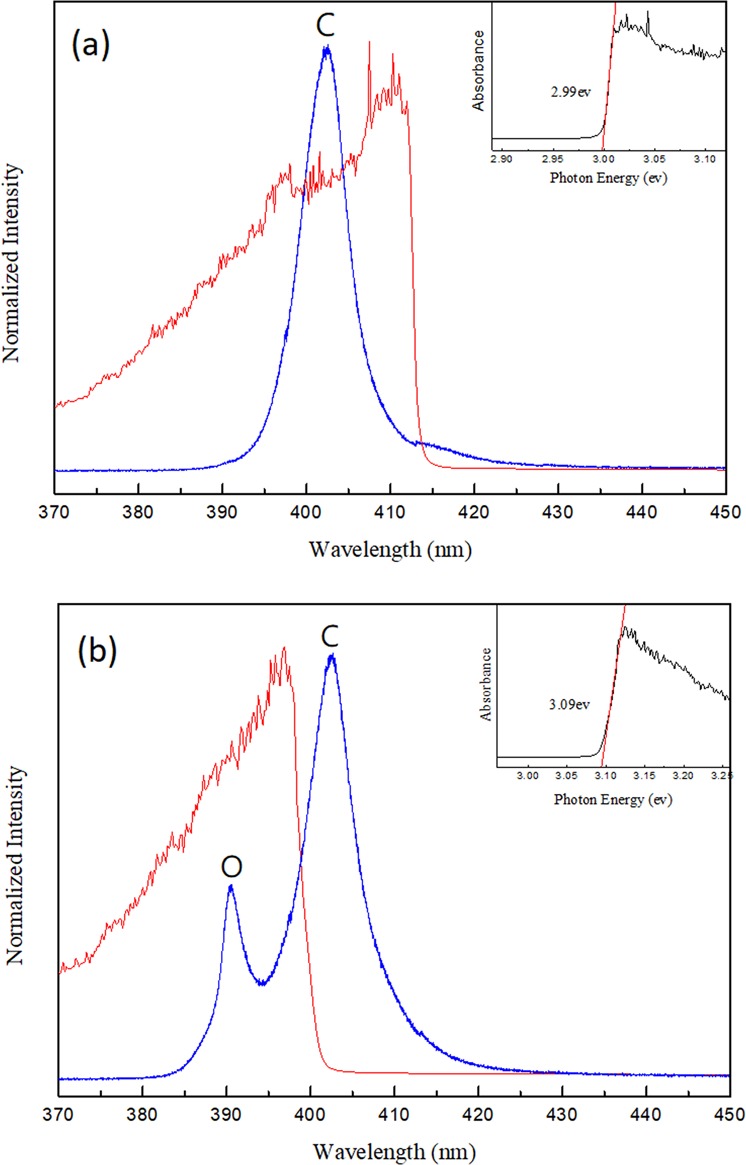
Absorption spectra of MAPbCl_3_ single crystals excited at 266 nm at (**a**) 180 K and (**b**) 170 K. C and O represent the emission from crystals in cubic and orthorhombic phases, respectively. The insets display the Tauc-plot fitting of absorption edges.

The structure changes were also manifested in the absorption and PL spectra. Figure 8(a) displays the temperature dependent PL spectra recorded from 190 K to 170 K, in which the PL line shape dramatically changed at ~175 K. Different from the previous temperature stage, three emission peaks (Fig. 8(b)) are required to fit the representative PL spectrum recorded at 175 K. Other than the peaks 1 and 2, which were ascribed to the co-existing cubic phase transitions and Cl vacancy^33^, a third peak (peak 3) appeared at ~390 nm. This newly emerged peak 3 not only persisted but also continuously grew in intensity when the temperature was further cooled down below 170 K. The presence of the emission peak 3 at temperatures under 175 K was most likely induced by the secondary phase transition, i.e. transition from tetragonal to orthorhombic. The shift in the absorption edge was validated by measuring the temperature dependent absorption spectra. The absorption edge displayed a significant blue shift (from 2.99 eV to 3.09 eV) when the temperature was lowered from 180 K to 175 K as shown in Fig. 9(a,b). We were not able to resolve the first phase transition (from cubic to tetragonal) in the PL/absorption spectra due to the limited temperature resolution (>5 K) and because these two phase transitions were only separated by ~6 K in temperature^51^. Moreover, another new peak 4 appeared at ~395 nm and persisted even at temperature below 170 K when the PL spectrum measured at 170 K was fitted (as shown in Fig. 8(c)). We speculated that this peak was also originated from the Cl vacancy in orthorhombic phase.

### 160 K to 20 K

**Figure 10:**
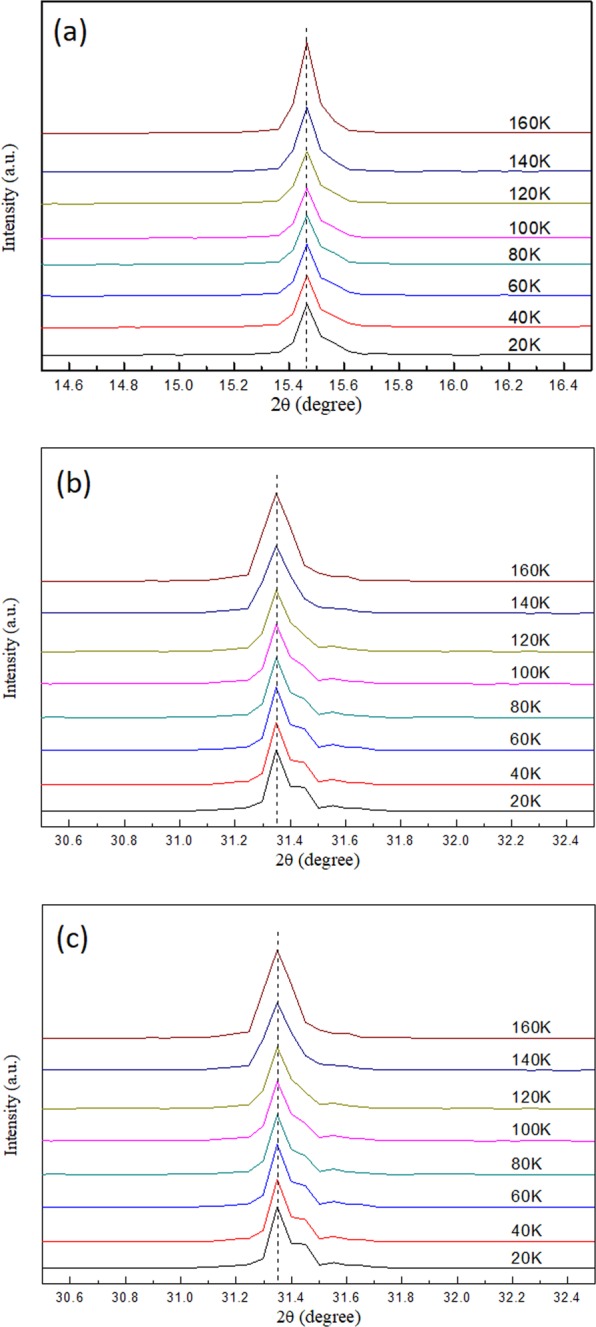
Diffraction spectrum of (**a**) (100) (**b**) (200) and (**c**) (300) peak from single crystal XRD measurements of MAPbCl_3_ at 160–20 K.

**Figure 11:**
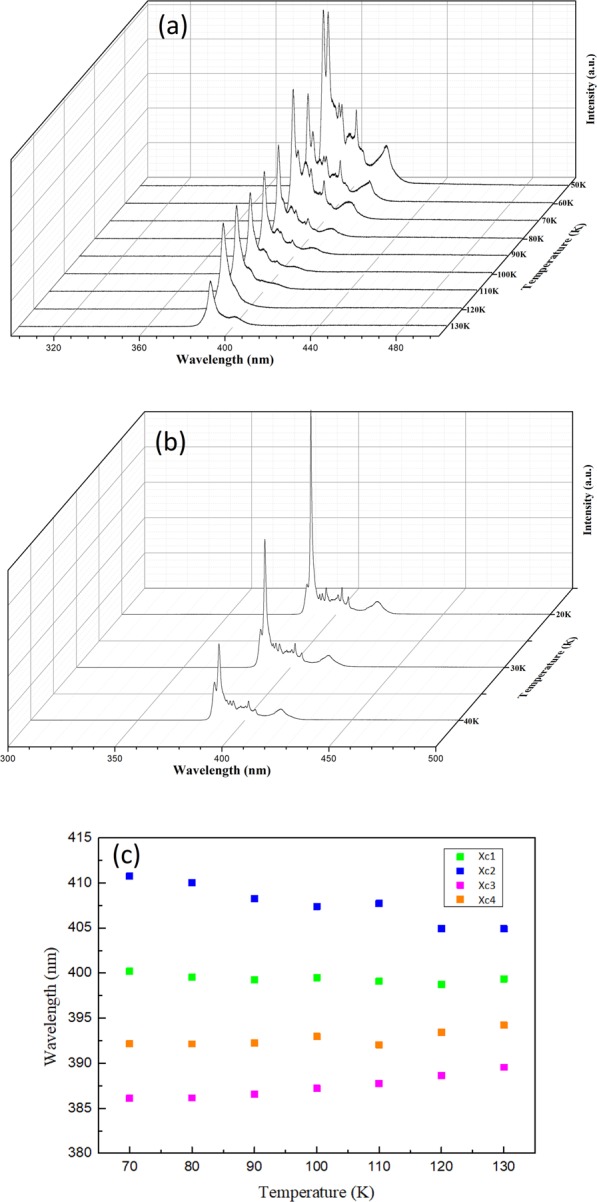
PL spectra of MAPbCl_3_ single crystals excited at 266 nm at (**a**) 130–50 K (**b**) 40–20 K. (**c**) Peak position as a function of temperature from 130 K to 70 K of peaks 1 to 4 from the fitting in Fig. 8(c).

**Figure 12:**
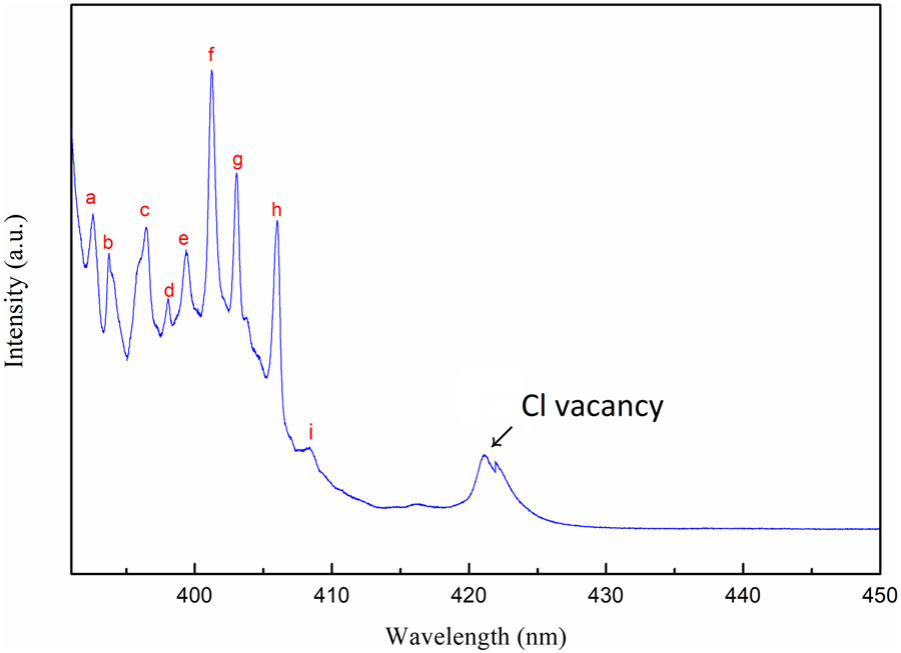
Representative PL spectrum between 390 nm and 450 nm at 20 K with excitation at 266 nm. The sharp spectral lines are labelled from a to i. The peak at ~422 nm is due to the Cl vacancy.

The temperature dependent single crystal XRD patterns of the crystal planes (100), (200), and (300) obtained from 160 K to 20 K are shown in Fig. 10. No noticeable shift in diffraction angle and change in line shape can be observed, thereby implying that the crystal remained relatively stable in orthorhombic phase throughout this temperature stage. The PL spectra obtained from 130 K to 20 K are displayed in Fig. 11(a,b). At the initial cooling stage, most PL peak positions remained nearly unchanged except peak 2, which moved toward lower energy as the temperature dropped (as shown in Fig. 11(c)). In addition to the increasing in emission intensity, the PL line shape also dramatically changed between 390 and 410 nm when the temperature dropped below 90 K. Several sharp spectral lines gradually emerged during cooling, as shown in Fig. 11(b,c). The representative PL spectrum with labelled sharp spectral lines (peak a to peak i) between 390 and 450 nm at 20 K is displayed in Fig. 12. The offset energy of the sharp lines relative to the band edge of the orthorhombic phase (peak 3) at 20 K was measured and is summarized in Table 2. The measured offset energy had the same order as the vibrational/librational modes of the cation and metallic frame. Therefore, we carefully crosschecked and compared these values with the results reported in ref. ^42^, in which Raman bands of the polycrystalline MAPbCl_3_ samples were recorded in orthorhombic phase and the associated translational and librational motions of different groups were assigned. We uncovered that the energies of these sharp spectral lines corresponded to either a single or a combination of the translational and/or librational motions of MA^+^. This finding has never been reported in other lead halide perovskites, such as MAPbI_3_ or MAPbBr_3_. We speculate that the MAPbCl_3_ single crystal has stronger polar nature and dipole momentum because of the enhanced polarization between PbCl_6_^−^ and MA^+^ due to the higher electron negativity of the chloride atoms. This result is relatively similar to those for inorganic polar semiconductors (such as GaAs). Therefore, vibronic coupling occurred between the photoexcited carriers and the translational and librational motions from the MA groups, and this phenomenon was more enhanced in MAPbCl_3_. With decreasing temperature, the intensity and FWHM of those vibronic replicas became strong and narrow due to the long translational/librational mode dephasing time. It will be interesting to further investigate the dynamics of these vibronic replicas.

**Table 2:** The measured offset energy of sharp spectral lines at 20 K shown in Fig. 12 and their assignments to the translational/librational modes of MA^+^.

Peak label	Energy offset ΔE (meV)	Peak assignment (ref. ^42^)
a	29.5	T’/R’ of MA^+^ (238 cm^−1^ ~29.5 meV)
b	38.84	T’/R’ of MA^+^ (321 cm^−1^ ~39.8 meV)
c	60.49	MA^+^ torsion (483 cm^−1^ ~60 meV)
d	76.39	T’/R’of MA^+^(321 cm^−1^ ~39.8 meV) + T’/R’of MA^+^(321 cm^−1^ ~39.8 meV)
e	86.54	T’/R’ of MA^+^(238 cm^−1^ ~29.5 meV) + MA^+^ torsion (483 cm^−1^ ~60 meV)
f	100.32	T’/R’ of MA^+^ (321 cm^−1^ ~39.8 meV) + MA^+^ torsion (483 cm^−1^ ~60 meV)
g	115.10	MA^+^ rocking (923 cm^−1^ ~114 meV)
h	137.39	C-N stretch (977 cm^−1^ ~121 meV) + T’/R’ of MA^+^ (119 cm^−1^ ~14.7 meV)
i	155.33	C-N stretch (1263 cm^−1^ ~156 meV)

In the end, we studied the electronic characteristics of the MAPbCl_3_ single crystal using I–V and Hall measurements. The schematics of the devices are displayed in the inset of Figs S6 and S7. The as-grown MAPbCl_3_ single crystal was determined as an n-type semiconductor with a carrier concentration of ~2.63 × 10^11^ cm^−3^ and a mobility of ~4.14 cm^2^/V•s according to the Hall measurements as shown in Fig. S6. The lower carrier mobility of MAPbCl_3_ compared with that of MAPbI_3_ and MAPbBr_3_^52^ is due to the stronger ionic nature of the Pb-Cl bonding. Figure S7(a,b) display the I–V curves of the crystal under dark and illumination. The I–V responses showed linear behavior with scanned bias from 0–40 V. A conductivity of ~1.8 × 10^−8^ *Ω*^−1^*cm*^−1^ was estimated and was in good agreement with earlier reports^36^. When illuminated with a 405 nm laser, the conductivity of the crystal increased by approximately an order of magnitude to ~9.7 × 10^−8^ *Ω*^−1^*cm*^−1^ as shown in Fig. S7(b).

## Conclusions

The crystal structures, photophysical and electronic characteristics of MAPbCl_3_ single crystals were studied using temperature dependent XRD, optical spectroscopy techniques, I–V, and Hall measurements. The crystal went through two phase changes, namely, cubic to tetragonal (at ~180 K) and tetragonal to orthorhombic (at ~175 K) when the temperature decreased from 300 K to 20 K. The crystal surface was sensitive to the environment, thereby implying that MAPbCl_3_ perovskites can be utilized as gas sensors. In contrast to our earlier studies on MAPbBr_3_ single crystals^33^, vibronic replicas were observed for the first time at temperatures under 90 K because of the higher electron negativity of chloride atom that led to the stronger polar nature of the crystal. This result suggests that, unlike the other organo-lead halide-based perovskites, the photoexcited carriers are able to interact with the organic cations and transfer their energy into vibrational/librational motions of organic molecules. The pristine crystal was demonstrated as an n-type semiconductor at 300 K with a carrier concentration of ~2.63 × 10^11^ cm^−3^, a mobility of 4.14 cm^2^/V•s, and a conductivity of 1.8 × 10^−8^ *Ω*^−1^*cm*^−1^ under dark conditions. The conductivity increased by approximately an order of magnitude when it was illuminated with a 405 nm laser. Our new findings can help future development of using MAPbCl_3_ perovskites for UV optoelectronics.

## Supplementary information

Structural, Photophysical, and Electronic Properties of CH3NH3PbCl3 Single Crystals
