# Supplementary material for: Structural, Photophysical, and Electronic Properties of CH3NH3PbCl3 Single Crystals
Source: Sci Rep. 2019 Sep 16;9:13311. doi: 10.1038/s41598-019-49926-z (PMC6746810; doi:10.1038/s41598-019-49926-z)
Supplement: Supplementary file 1 — Structural, Photophysical, and Electronic Properties of CH3NH3PbCl3 Single Crystals [file 41598_2019_49926_MOESM1_ESM.pdf]

Supporting Information

Structural, Photophysical, and Electronic Properties of $CH_3NH_3PbCl_3$ Single Crystals

Hao-Ping Hsu[1], Liang-Chen Li[3], Muthaiah Shellaiah[1], and Kien Wen Sun[1,2,3]*

[1]Department of Applied Chemistry, National Chiao Tung University, 1001 University Road, Hsinchu 30010, Taiwan
[2]Department of Electronics Engineering, National Chiao Tung University, 1001 University Road, Hsinchu 30010, Taiwan
[3]Center for Nano Science and Technology, National Chiao Tung University, 1001 University Road, Hsinchu 30010, Taiwan

Table of contents:

SEM and TEM images of $MAPbCl_3$ single crystals **(S3)**

XRD patterns of $MAPbCl_3$ single crystals measured at 300-20 K **(S4)**

Temperature-dependent PL spectra at 300-20 K **(S5)**

Illustraion of the structural changes at different temperature stages **(S6)**

Temperature-dependent plane spacing of the (100) crystal planes **(S7)**

Hall measurements of $MAPbCl_3$ single crystal (**S8**)

Current-Voltage characteristics of $MAPbCl_3$ single crystal (**S9**)

(a)

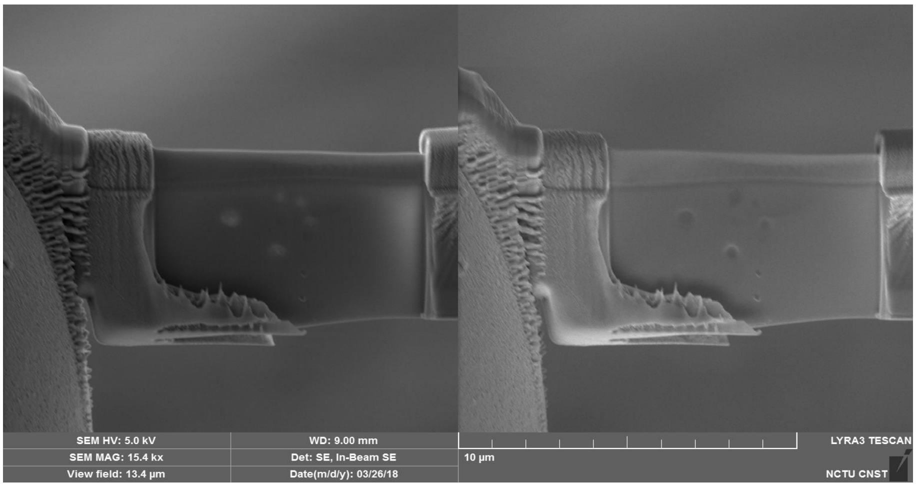

(b)

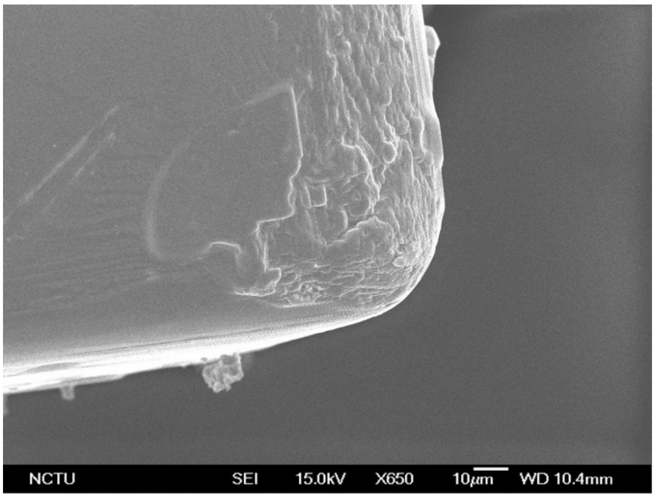

(c)

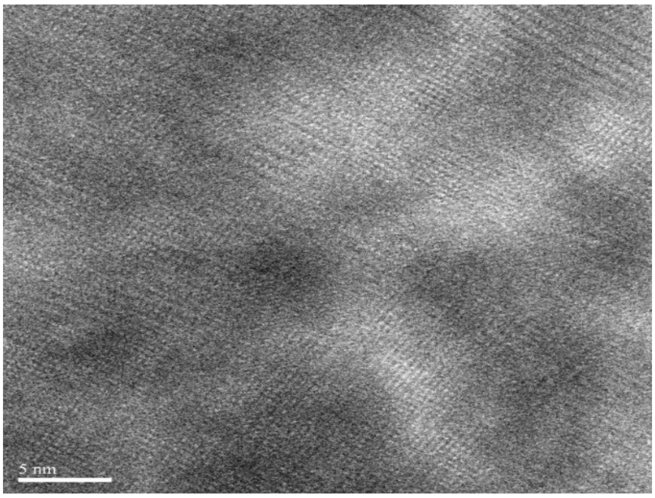

Figure S1. (a) SEM image of a thin slice cut from $MAPbCl_3$ single crystals using focus ion beam. Note that several dents were created near the image center during the ion beam milling processes. (b) SEM and (C) TEM images of $MAPbCl_3$ single crystal powder. Amorphization or liquidation of the samples immediately occurred during the observation.

(a)

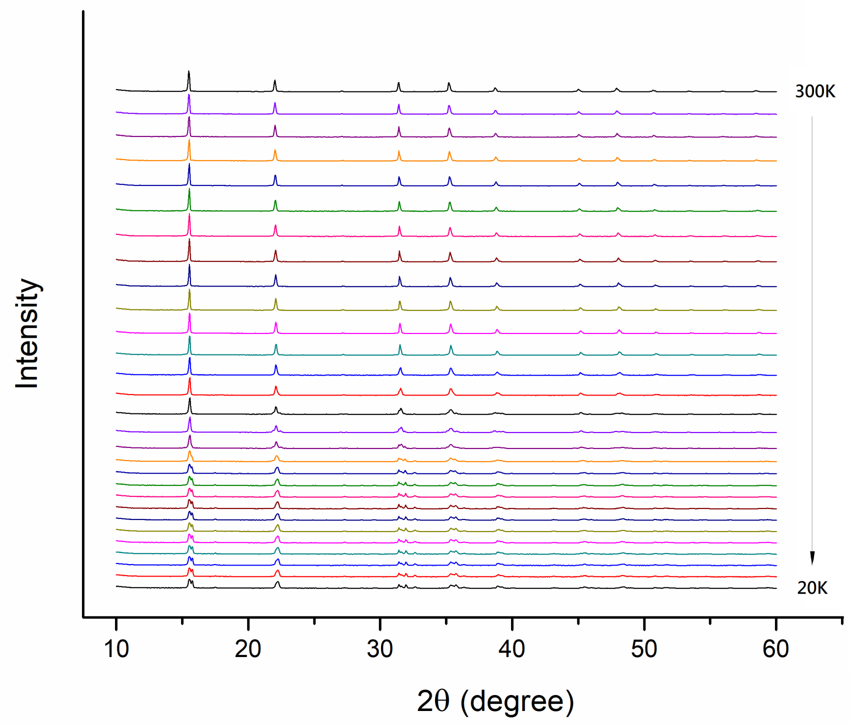

(b)

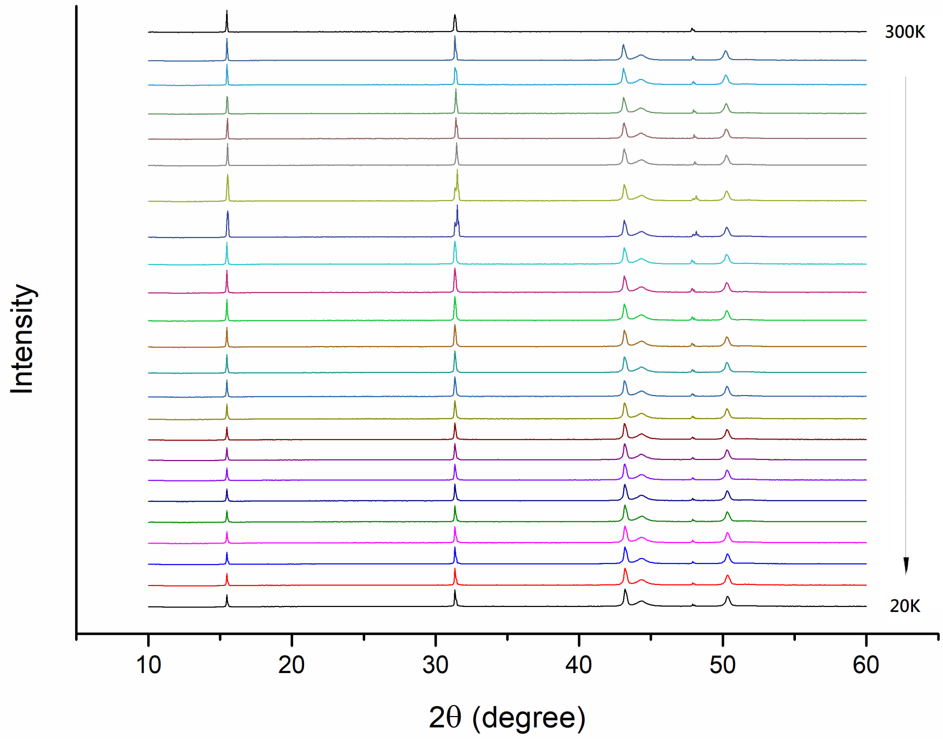

Figure S2. (a) Powder and (b) single-crystal XRD patterns of $MAPbCl_3$ single crystals measured at 300-20 K.

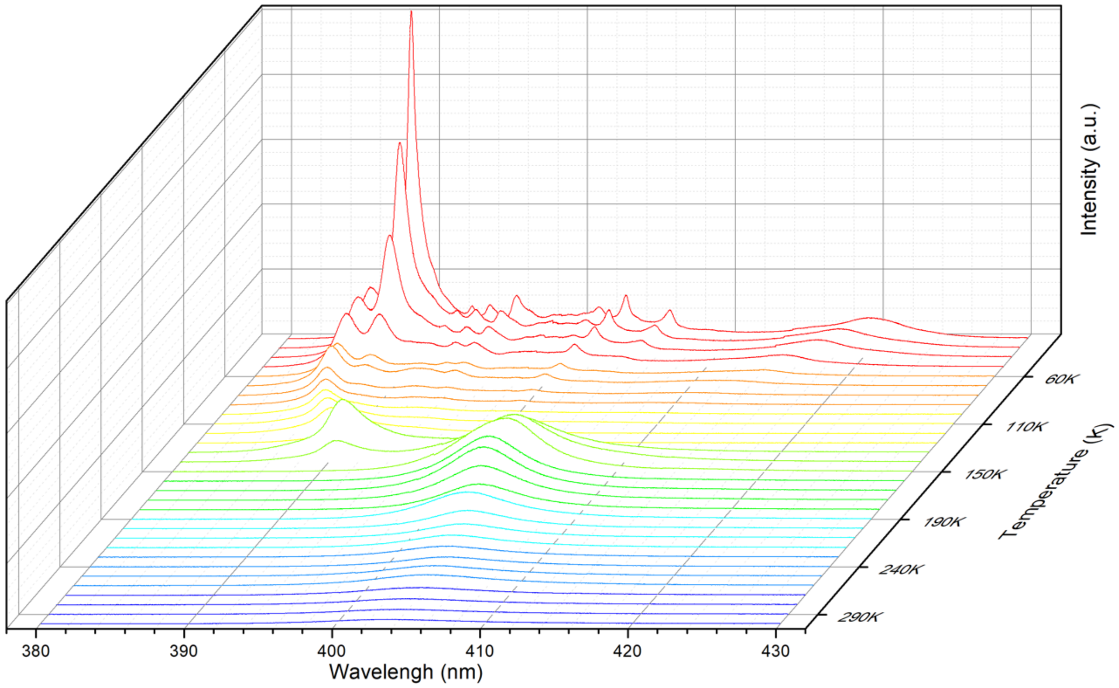

Figure S3. Temperature-dependent PL spectra of $MAPbCl_3$ single crystals measured at 300-20 K.

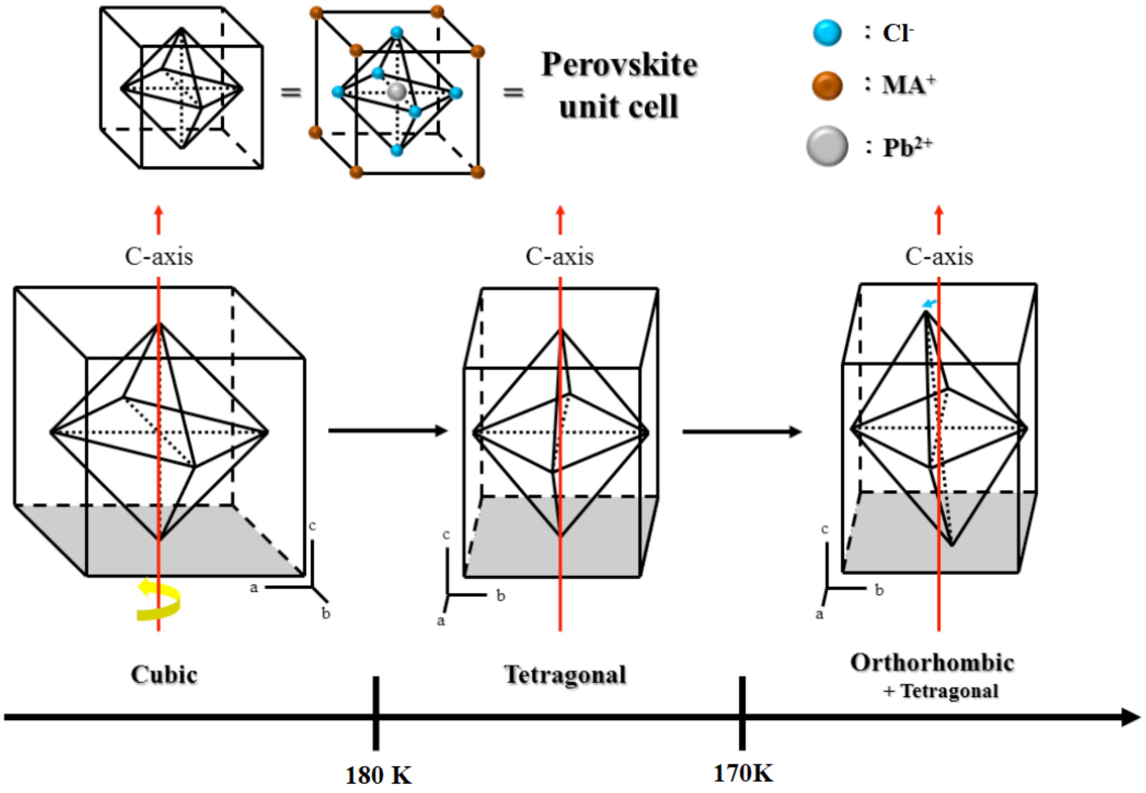

Figure S4. Structural changes at different temperature stages throughout the cooling process are displayed through a schematic of the 3D $MAPbCl_3$ crystal structure.

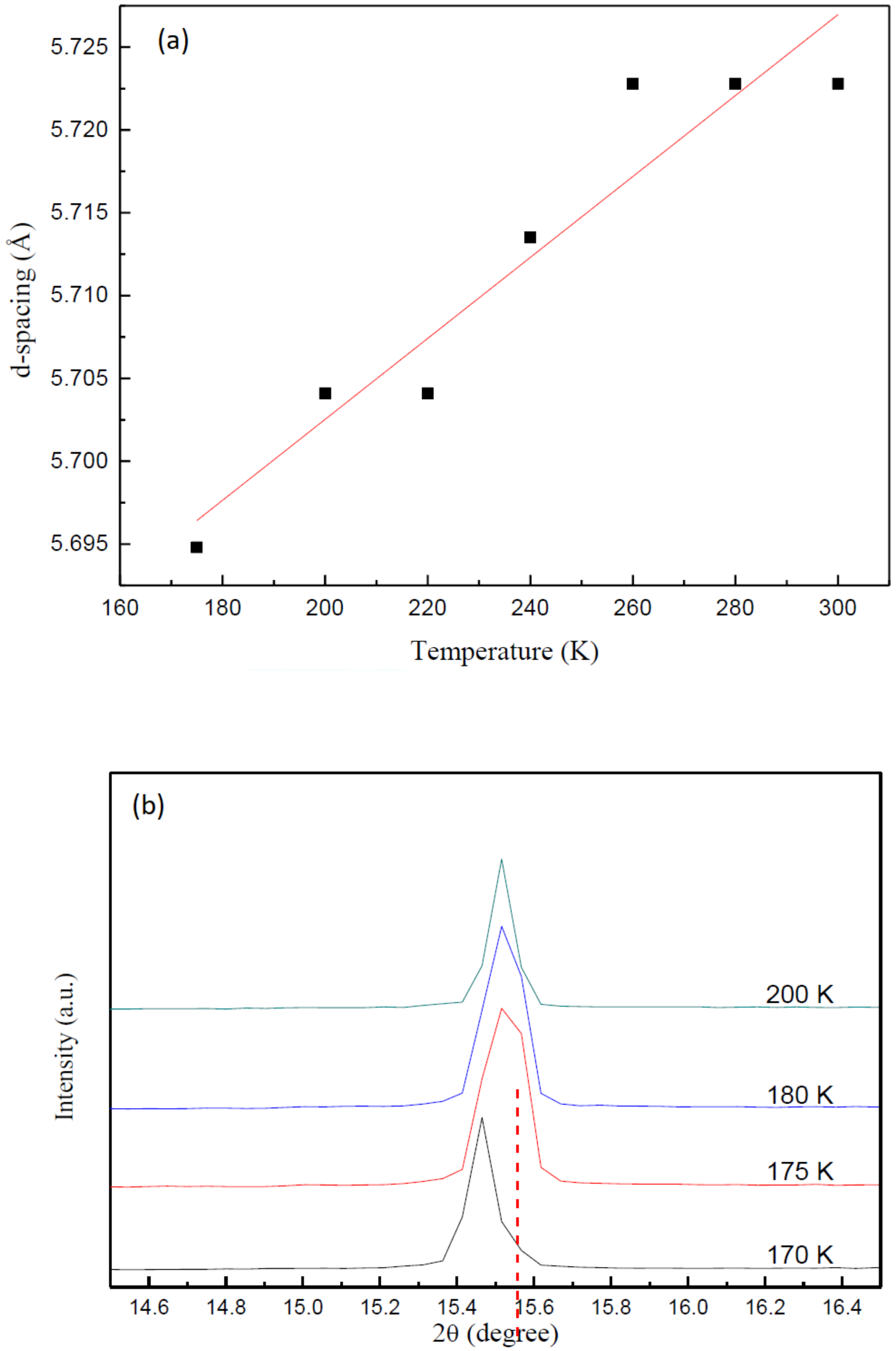

Figure S5. (a) Calculated temperature-dependent d-spacing of the (100) crystal planes in cubic phase. (b) Temperature-dependent XRD of the single crystal from 200-170 K. The red dashed line marked the predicted (100) diffraction angle at 170 K using the thermal expansion coefficient retrieved from (a).

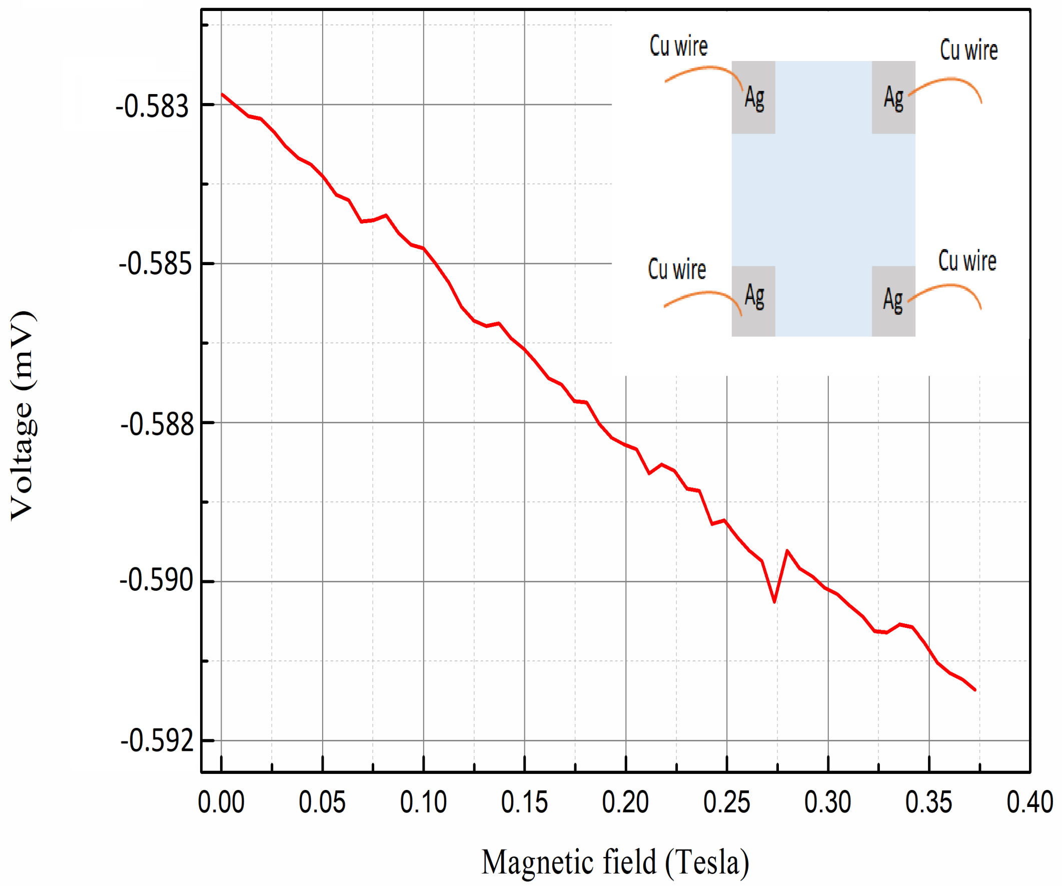

Figure S6. Hall voltages as a function of scanned magnetic field up to 0.375 Tesla. Inset displays the schematic of the $MAPbCl_3$ single crystal Hall device.

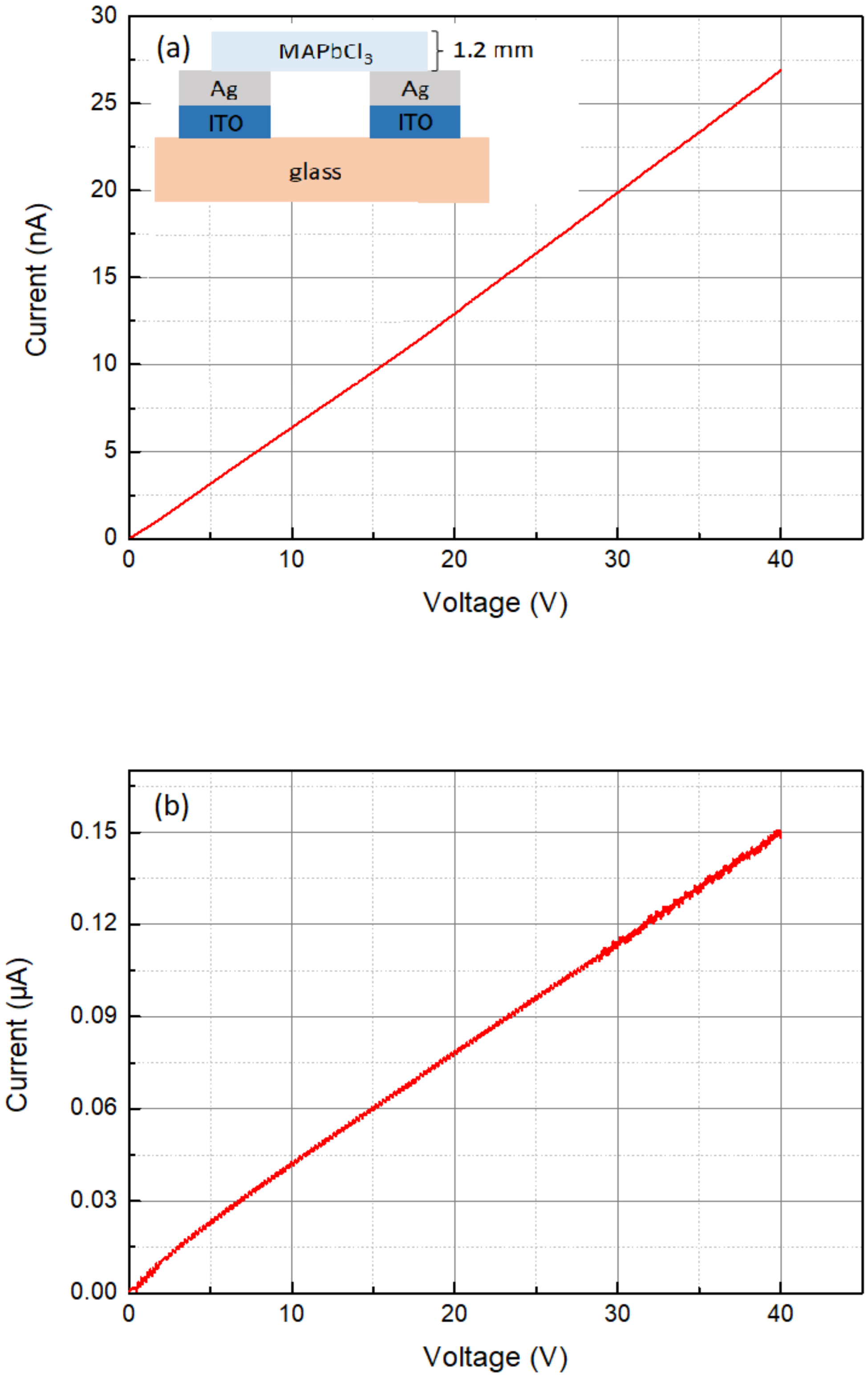

Figure S7. I-V characteristics of the $MAPbCl_3$ crystal (a) under dark (b) illuminated with a 405 nm laser. Inset in (a) displays the schematic of the device for the I-V tests.
